# Dectin-1 participates in the immune-inflammatory response to mouse *Aspergillus fumigatus* keratitis by modulating macrophage polarization

**DOI:** 10.3389/fimmu.2024.1431633

**Published:** 2024-10-16

**Authors:** Liu Guibo, Dong Chunxu, Chen Biao, Hu Zhaolei, Liu Wenwen, Ji Xiangnan, Peng Wentao, Chang Hongmin, Li Yonghua, Zhu Guoqiang

**Affiliations:** ^1^ Department of Ophthalmology, Affiliated Hospital of Jining Medical University, Jining, Shandong, China; ^2^ Department of Ophthalmology, Qingdao University, Qingdao, Shandong, China; ^3^ Department of Ophthalmology, Jining Medical University, Jining, Shandong, China; ^4^ Department of Ophthalmology, Jiaozhou Central Hospital, Qingdao, Shandong, China

**Keywords:** fungal keratitis, dectin-1, innate immune response, macrophage, polarization

## Abstract

**Aim:**

The aim of this study was to investigate whether Dectin-1 influences the immune-inflammatory response in *A. fumigatus* keratitis by modulating macrophage polarization.

**Methods:**

1. The models of 1-day, 3-day, and 5-day of fungal keratitis were established in SPF C57BL/6 mice after stimulation by *A. fumigatus.* Dectin-1 agonist (curdlan) and antagonist (laminaran) were injected separately in the mouse subconjunctivae for 1 day in the established mouse model of *A. fumigatus* keratitis; PBS was used as the control. Inflammation of the mouse cornea was observed under a slit lamp to obtain a clinical score. 2. The expression of M1 (TNF-α, INOS, IL-6, IL-12) and M2 (Arg-1, IL-10, Fizz-1, Ym-1) cytokine-encoding mRNAs was quantified by RT-PCR. 3. Changes in the number of macrophages and expression of M1 and M2 macrophages in mouse corneas detected by immunofluorescence and flow cytometry. 4. Pre-treatment of RAW264.7 cells with MAPK cell signaling pathway inhibitors SB203580 (p38 inhibitor, 10µM), U0126 (ERK inhibitor, 20µM), SP600125 (JNK inhibitor, 10µM) and DMSO separately for 2 h, and stimulated by *A. fumigatus* for 12 h. Changes in the mRNA expression of M1 and M2 cytokines in the macrophages were quantified by RT-PCR.

**Results:**

1. With curdlan pre-treatment, mouse corneal inflammation worsened, and the clinical score increased after infection. In contrast, in the laminaran pre-treated group, corneal inflammation was alleviated and the clinical score decreased significantly compared to the PBS group after infection. 2. Compared with the control group, the expression levels of macrophage phenotype-related M1 and M2 cytokine mRNAs increased significantly 1, 3, and 5 days after *A. fumigatus* infected the corneas of mice. 3. With curdlan pre-treatment, the expression of mRNAs encoding M1 cytokines increased, while those encoding M2 cytokines decreased in the cornea compared to the PBS group. In contrast, after infection, mRNA levels for M1 cytokines decreased significantly and those for M2 cytokines increased in the cornea of the laminaran pre-treated group compared to the PBS group. 4. The number of macrophages in the corneal stroma of mice in the curdlan pretreatment group increased significantly compared with the PBS group, while in the laminaran pretreatment group this number decreased significantly. 5. The results of flow cytometry showed that after 3 days of mouse corneal *A. fumigatus* infection, the number of macrophages in the mouse *A. fumigatus* model in the curdlan pretreatment group was increased (10.4%) and the number of macrophages in the mouse *A. fumigatus* model in the laminaran pretreatment group (6.31%), when compared with the AF+FBS group (7.91%). The proportion of M1-type macrophages was increased in the curdlan pretreated group (55.6%) compared to the AF+FBS group (51.2%), the proportion of laminaran pretreatment group had a decreased proportion of M1-type macrophages (46.8%); while M2-type macrophages were the opposite of M1-type: the proportion of M2-type macrophages was 49.2% in the AF+FBS group, the proportion of M2-type macrophages was decreased in the curdlan pretreatment group (44.0%), and the proportion of M2-type macrophages was increased in the laminaran pretreatment group (53.5%). 6. Expression of M1 and M2 cytokine-encoding mRNAs decreased and increased, respectively, after infection, in the RAW264.7 cells pre-treated with MAPK pathway inhibitors, compared to the control.

**Conclusion:**

In a mouse model of *A. fumigatus* keratitis, Dectin-1 can affect macrophage recruitment and polarization, may regulate macrophage phenotype-associated factor changes through the MAPK signaling pathway.

## 1 Introduction

Fungal keratitis is a serious corneal infection and severe blinding eye disease in rural areas of developing countries (1). In China, corneal injuries from plant scratches during agricultural activities are a leading cause of fungal keratitis. With the rising number of people wearing contact lenses, the incidence of this condition has been increasing annually (2). The most common pathogens are filamentous fungi such as *Fusarium* and *Aspergillus fumigatus* (3). The cornea is the first line of defense against ophthalmic fungal infection (4). Innate immunity triggers an immune inflammatory response by recognizing pathogen-related molecular patterns on pathogenic microorganisms through pattern recognition receptors. Dectin-1 is a pattern recognition receptor belonging to the family of C-type lectin receptors. It is highly expressed in dendritic cells, mononuclear phagocytes, neutrophils, and other cells (5, 6). Through specific recognition of, and binding to, β-glucan in fungal cell walls, Dectin-1 can generate large amounts of reactive oxygen species and cytokines through the inflammatory signaling cascade. It can also guide inflammatory cells to sites of inflammation through chemotaxis, thereby playing an important role in the anti-fungal process in the cornea (3, 7).

Macrophage are one of the important components of the body’s innate immune system. They have functions of phagocytosis, antigen presentation, and secretion of a variety of cytokines (8, 9). Macrophage activation is described as a continuum, with different stimuli inducing M1, M2, or mixed phenotypes. M1-like macrophages highly express CD68, CD80, CD86, major histocompatibility complex (MHC)-II, inducible nitric oxide synthase (INOS), and Toll-like receptor (TLR). It can be activated by Th1 cytokines (TNF-α and IFN-γ) alone or in conjunction with pathogen-associated molecular patterns (e.g., LPS). M2-like macrophages express high levels of c-type lectins (CD206) and endocytic receptors (CD163). It can be activated by Th2 cytokines (IL-4 and IL-13), TGF-β, IL-10, glucocorticoids, and immune complexes (10–12). When pathogens invade the body, macrophage can be polarized into the M1 type due to changes in the microenvironment. At the same time, these inflammatory factors can induce macrophage apoptosis or drive polarization toward the M2 type, which helps alleviate inflammation, reduce excessive damage, and promote healing (14–18).

Mitogen-activated protein kinases (MAPKs) are a family of intracellular serine/threonine protein kinases that are important signal transduction pathways in eukaryotic cells (19). Dectin-1 can activate MAPKs through the downstream spleen tyrosine kinase (Syk) signaling pathway during fungal keratitis (20, 21). At the same time, the MAPK signaling pathway (including P38, c-Jun N-terminal kinase (JNK), and extracellular signal-regulated kinase (ERK)) was found to participate in generating macrophage phenotype-related factors in a macrophage polarization model (20, 22–24). Previously, it has been shown that p38 participates in the transformation of M1 to M2 during tissue repair and regulates the expression of phenotype-related factors in macrophages (25).

However, it is still unclear whether Dectin-1 regulates macrophage function and its mechanism in *Aspergillus fumigatus* keratitis. Considering the important role of Dectin-1 and its macrophages in fungal keratitis. Therefore, we investigated the effect of Dectin-1 on macrophage polarization and its potential mechanism in *Aspergillus fumigatus* keratitis.

## 2 Materials and methods

### 2.1 Culture of *A. fumigatus* and preparation of the infection solution

Inoculate *A. fumigatus* (the standard strain, no. 3.0772, was purchased from the China General Microbiological Culture Collection Center) spores onto Sabouraud medium on a clean work-bench. When *A. fumigatus* developed clumpy hyphae, the hyphae were isolated, ground, treated with 75% ethanol for inactivation, and washed three times with sterile phosphate-buffered saline (PBS). The final concentration of hyphae was adjusted to 1 × 10^8^/colony forming units with a counting plate.

### 2.2 Mouse fungal keratitis model

Healthy female C57BL/6 mice, 8 weeks of age, were obtained from Chinese committee for the preservation of microbial species. Mice were in good general condition, and any with eye diseases were excluded. In total, 8 of 100 mice were excluded for failure to meet these criteria (corneal abrasion, n = 5; eyeball atrophy, n = 2; anophthalmia,n = 1). Mice were anesthetized by intraperitoneal injection of 8% chloral hydrate (0.4 mL/Kg). The central corneal epithelium of both eyes was scraped with a microkeratome to create a defect area. The defect area in the right eye was smeared with an inactivated *A. fumigatus* fungal solution (1 × 10^8^/colony forming units). Thereafter, the eye surface was covered with soft contact lenses and the eyelids were sutured. The left eye was used as the blank control. Both eyelids were then sutured shut. All animal handling processes conformed to the Chinese Ministry of Science and Technology Guidelines on the Humane Treatment of Laboratory Animals (vgkfcz-2006-398), and the principles and standards of the United States Association for Research in Vision and Ophthalmology for the use of animals in ophthalmic and visual research.

### 2.3 Measurement of macrophage phenotype-related factors after induction of *A. fumigatus* keratitis

The eyelid sutures were opened at 1, 3, and 5 days after treatment, and changes of corneal inflammation were observed under a slit lamp microscope. The mice were then sacrificed and full corneas of experimental and control eyes were collected. RNAiso plus reagent was used to separate RNA from the suspension. Then, spectrophotometry was used to quantify RNA obtained at a fast speed. For reverse transcription, we used 1 μg RNA for the first-strand cDNA synthesis. Then, based on the manufacturer’s instructions, we used 2 μL cDNA for polymerase chain reaction (PCR) in a 20 μL reaction volume. Nucleotide sequences of mouse primers for real-time RT-PCR (**Table 1**).

**Table 1:** Nucleotide sequences of mouse primers for real-time RT-PCR.

TNF-α	NM_013693.2	F: ACC CTC ACA CTC AGA TCA TCT T
		R: GGT TGT CTT TGA GAT CCA TGC
INOS	NM_010927.3	F: TGT CTG CAG CAC TTG GAT CAG
		R: AAA CTT CGG AAG GGA GCA ATG
IL-6	NM_008361.3	F: CAC AAG TCC GGA GAG GAG AC
		R: CAG AAT TGC CAT TGC ACA AC
IL-12	NM_008352.2	F: GGG ACC AAA CCA GCA CAT TG
		R: TAC CAA GGC ACA GGG TCA TCA
Arg-1	NM_007482.3	F: TGG GTG ACT CCC TGC ATA TCT
		R: TTC CAT CAC CTT GCC AAT CC
IL-10	NM_010548.2	F: TGC TAA CCG ACT CCT TAA TGC
		R: TTC TCA CCC AGG GAA TTC AAA
Fizz-1	NM_020509.4	F: TCC CAG TGA ATA CTG ATG AGA
		R: CCA CTC TGG ATC TCC CAA GA
Ym-1	NM_0009892.4	F: GGG CAT ACC TTT ATC CTG AG
		R: CCA CTG AAG TCA TCC ATG TC

### 2.4 Pretreatment of mice with the dectin-1 agonist, curdlan, or inhibitor, laminaran

In the Dectin-1 stimulation and inhibition experiments, 5μL of curdlan (1.5 mg/mL) or laminaran (1 mg/mL) were injected subconjunctivally one day before *A. fumigatus* infection. Determination of concentration and dosage based on combination of some articles previously published in our laboratory for experiments involving dectin-1 agonist, curdlan, inhibitor, or laminarin concentrations. Our laboratory has previously found that Dectin-1 agonist curdlan modulates innate immunity to *Aspergillus fumigatus* in human corneal epithelial cells (26). The concentration of the Dectin-1 inhibitor laminarin is determined based on articles published by Shi and colleagues from our laboratory (27). Five microliters of PBS were injected subconjunctivally into the control eyes. Three days after *A. fumigatus* infection, corneal infection was observed under a microscope and corneal photos were taken. The keratitis reaction of mice was observed under a slit lamp and the clinical score was recorded. RT-PCR was used to detect changes in the expression of macrophage M1 and M2 cytokine mRNAs in the corneas of mice pretreated with the Dectin-1 agonist and inhibitor, and the PBS group.

### 2.5 Western blot analysis

The corneas were ground and dissolved in radioimmunoprecipitation lysis buffer. The proteins were then mixed with sodium dodecyl sulfate-polyacrylamide gel electrophoresis (SDS-PAGE) sample loading buffer and incubated for 10 minutes in a boiling water bath. Proteins were separated using a 12% SDS-PAGE in Tris/glycine/SDS buffer and subsequently transferred to a membrane. The membranes were blocked and then incubated overnight at 4°C with primary antibodies against INOS (1:500; Santa Cruz, CA, USA), ARG-1 (1:500; Santa Cruz, CA, USA), or β-actin (1:2000; Elabscience, Wuhan, China). Following this, the membranes were incubated at 37°C for 1 hour with an anti-rabbit secondary antibody (1:2000; Santa Cruz, CA, USA). Protein detection was performed using Beyo ECL Plus on an EC3 imaging system with Quantity One software.

### 2.6 Flow cytometry assay

After infecting mice corneas with *Aspergillus fumigatus* for 1, 3, and 5 days, we observed from the immunofluorescence results that the number of macrophages infiltrating the cornea was highest at 3 days post-infection (**Supplementary Figure 5S**). Therefore, we chose day 3 post-infection as the time point for macrophage flow cytometry analysis. Corneal tissue from each group of mice was collected the 3th day after Aspergillus infection and placed in EP tubes with sterile Hanks solution. Mouse corneal tissue was digested with Liberase for 2 hours to a single-cell suspension. Samples were then centrifuged, rinsed, re-centrifuged, blocked, flow antibodies added, and finally analyzed by Beckman flow cytometry and FlowJo X software. Using CD45+ as the expression gate, the expression of F4/80+, CD86+ and CD206+ in cells within the gate was further analyzed to analyze the macrophage number and M1/2 phenotype ratio. Primary antibodies were as follows CD45-PerCP, F4/80-PE-CY7, CD86-PE and CD206-FITC, all in the ratio of (1:200).

### 2.7 Inhibition of MAPK signaling pathways in *A. fumigatus-*exposed RAW264.7 macrophage

RAW 264.7 cells were pretreated with the p38 inhibitor, SB203580 (10 μM), JNK inhibitor, SP600125 (10 μM), or ERK inhibitor U0126-Etoll (20 μM) for 2 hours, then exposed to *A. fumigatus* for 12 hours. RT-PCR was used to detect changes in the expression of M1 and M2 cytokine mRNAs.

### 2.8 Immunofluorescence staining

Changes in the numbers of corneal macrophage were observed immunohistochemically. The eyeballs of mice in each group were removed, embedded in OCT compound, and quickly frozen in liquid nitrogen. Corneal tissues were cut into 10 µm sections with a freezing microtome, fixed in acetone for 10 min, washed three times with PBS, and drained. Tissues were then blocked with 10% goat serum (Solarbio China) for 20 min, incubated with a rat macrophage F4/80 antibody (Abcam, UK) at 37˚C for 2 h, and washed three times with PBS. A goat anti-rat FITC secondary antibody was then added at 37˚C for 1 h, washed with three times with PBS, and subjected to 4’,6-diamidino-2-phenylindole nuclear staining. Finally, the macrophage were observed under a fluorescence microscope.

### 2.9 Statistical analyses

All experiments were repeated three times to ensure the reproducibility and representative data were shown as mean ± SEM. SPSS 19.0 statistical software (IBM, USA) was used to analyze differences in the relative expression of mRNA. One-way and factorial analyses of variance were used for comparisons among groups. The least significant difference t test was used for comparison between two groups. P < 0.05 indicated that the difference was statistically significant.

## 3 Results

### 3.1 Effect of dectin-1 on the severity of the disease and clinical score of mouse fungal keratitis

**Figure 1:**
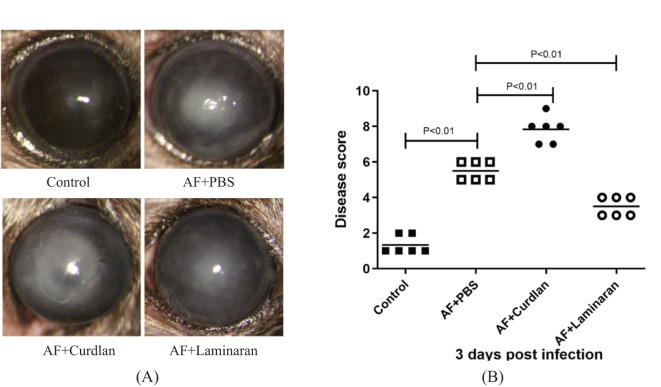
Disease response to Dectin-1 agonist or inhibitor treatment in *A*. *fumigatus* stimulated mice corneas. **(A)** Representative corneal images of *A*. *fumigatus* keratitis mouse models 3 days post infection after pretreatment of Dectin-1 agonist or inhibitor or PBS respectively. **(B)** Pretreated with Dectin-1 agonists had increased clinical scores 3 days after infection, and Dectin-1 inhibitors reduced clinical scores compared with pretreatment with PBS. Values represent as mean ± SEM. P<0.01 Mouse corneas (6/group).

The corneal inflammatory reaction of C57BL/6 mice infected by *A. fumigatus* after pretreatment with a Dectin-1 agonist or inhibitor was observed by a slit lamp microscope, and scored according to the clinical standard of fungal keratitis. Compared with the PBS group, pretreatment with the Dectin-1 agonist, curdlan, significantly increased the corneal ulcer area and depth, turbidity, and inflammation score (P < 0.01) at 3 days after fungal infection. In contrast, compared with the PBS group, mice pretreated with the Dectin-1 inhibitor, laminaran, had a significantly reduced corneal ulcer area and depth, turbidity, and inflammation score (P < 0.01) (**Figure 1**). These results suggest that Dectin-1 modulates corneal inflammation in C57BL/6 mice infected by *A. fumigatus*.

### 3.2 Changes in the expression of macrophage phenotype-related factors in mouse *A. fumigatus* keratitis

**Figure 2:**
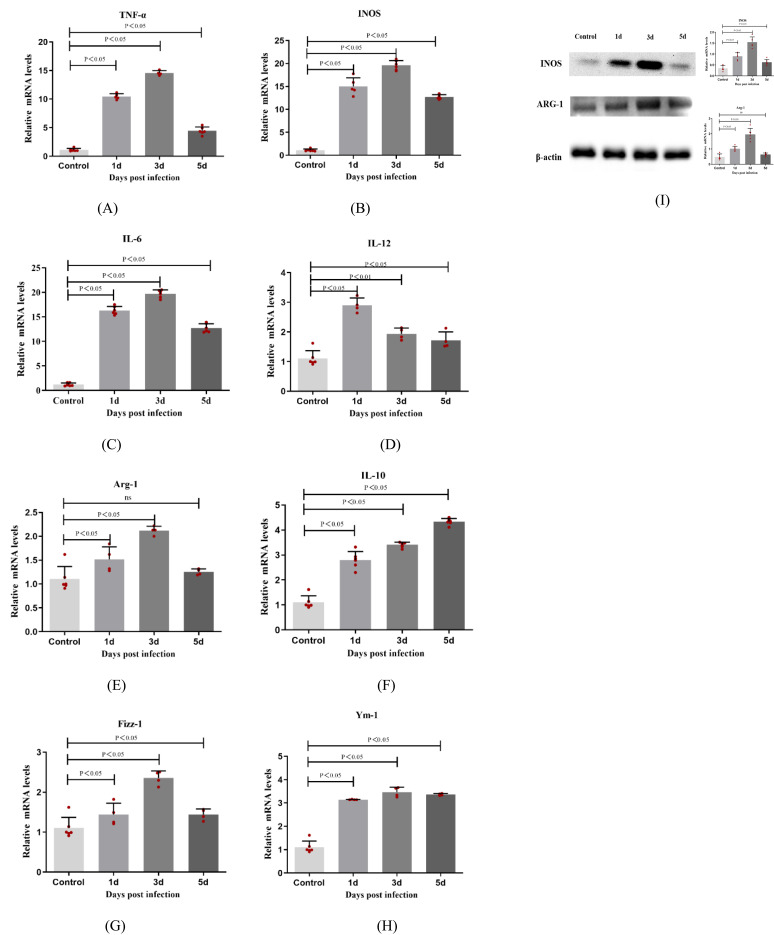
The expressions of TNF-α, INOS, IL-6, IL-12, Arg-1, IL-10, Fizz-1, and Ym-1 were examined by RT-PCR in mouse corneas following *A. fumigatus* infection at 1, 3, and 5 days **(A-H)**. Protein expression levels of INOS and ARG-1 in the infected corneas at 1, 3 and 5 days p.i. compared to the control group by Western Blot **(I)**. Values represent as mean ± SEM. Mouse corneas (6/group).

Compared with the control group, the expression levels of macrophage phenotype-related M1 (TNF-α, INOS, IL-6, and IL-12) and M2 (Arg-1, IL-10, Fizz-1 and Ym-1) cytokine mRNAs increased significantly 1, 3, and 5 days after *A. fumigatus* infected the corneas of mice (P < 0.05). The expression of M1 and M2 cytokines reached a peak on day 3 of infection and decreased on day 5 (**Figure 2**). The expression level of M1 macrophage phenotype related factor/M2 macrophage phenotype related factor INOS/ARG-1 protein is consistent with the trend of RT-PCR results. These results indicate that *A. fumigatus* can increase the expression of macrophage phenotype-related M1 and M2 cytokines in C57BL/6 mouse corneas.

### 3.3 Effect of dectin-1 on macrophage phenotype-related factors in mouse *A. fumigatus* keratitis

**Figure 3:**
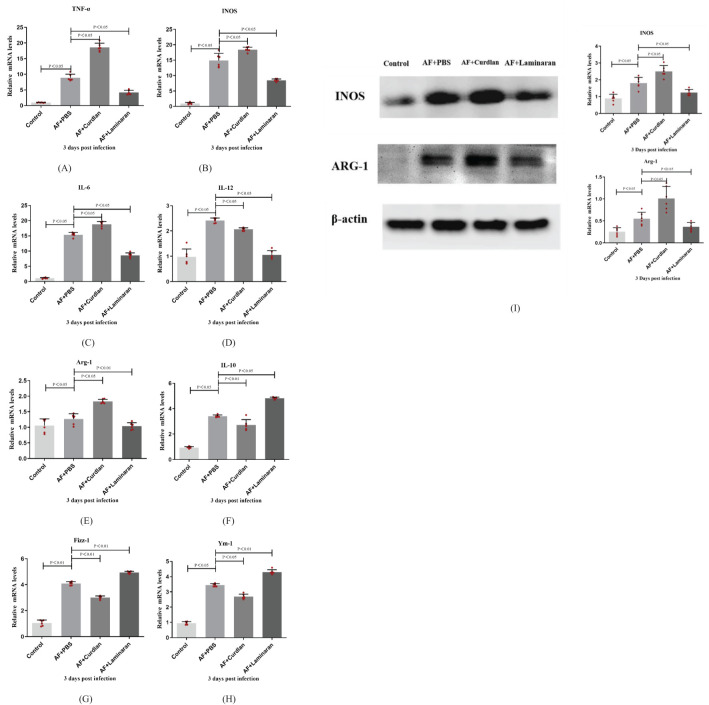
Effects of Dectin-1 agonist or inhibitor treatment on C57BL/6 mice corneas before infection and then infected with *A. fumigatus* for 3 days. The mRNA levels of TNF-α, INOS, IL-6, IL-12, Arg-1, IL-10, Fizz-1 andYm-1 were measured by RT-PCR **(A-H)**. The expression levels of INOS and Arg-1 proteins in the Dectin-1 agonist pretreatment group and inhibitor pretreatment group after corneal infection for 3 days were compared with the control group by Western Blot **(I)**.The values are expressed as mean ± SEM. Mouse corneas (6/group).

The cornea group of *A. fumigatus* uninfected mice was used as the control group. As shown in the analysis, compared with the PBS group, curdlan pretreatment elicited significantly higher mRNA expression levels of M1 (**Figures 3A–D**) and lower mRNA expression levels of M2 cytokines (**Figures 3E–H**) (P < 0.05). However, compared with the PBS group, the laminaran pretreatment group had significantly lower expression of M1 and significantly higher expression of M2 cytokines (P < 0.05). The expression level of M1 macrophage phenotype related factor/M2 macrophage phenotype related factor INOS/Arg-1 protein is consistent with the trend of RT-PCR results (**Figure 3I**). These results indicate that Dectin-1 can affect the expression of macrophage phenotype M1 and M2 cytokines in the corneas of C57BL/6 mice infected by *A. fumigatus*.

### 3.4 Effect of dectin-1 on macrophage recruitment in mouse *A. fumigatus* keratitis

**Figure 4:**
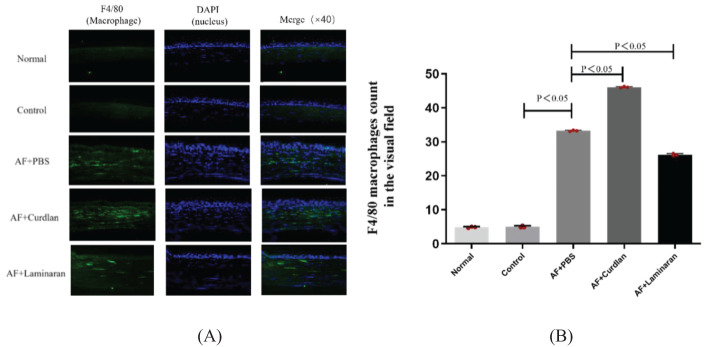
**(A)** Effect of Dectin-1 on macrophage infiltration in mice corneas. Mouse corneas were pretreated with Dectin-1 agonist or inhibitor or PBS before infection, then stimulate the corneas with *A. fumigatus* for 3 days. Infiltrating macrophage were directly observed and measured as a count per 10 µm cornea. F4/80 (green) and DAPI staining (blue). Magnification: 40X. **(B)** The comparison of macrophage count in the visual field of the corneas.

Macrophage-specific immunofluorescence staining showed that there was little or no macrophage infiltration in the corneas of normal C57BL/6 mice one day after a fungal infection when given a subconjunctival PBS pretreatment. By 3 days after *A. fumigatus* infection, a large number of macrophages infiltrated into the corneal stroma layer; there was a significant increase compared with the normal group (**Figure 4**). The number of macrophages in the corneal stroma of mice in the curdlan (Dectin-1 agonist) pretreatment group increased significantly compared with the PBS group, while in the laminaran (Dectin-1 inhibitor) pretreatment group this number decreased significantly. These results suggest that Dectin-1 may promote the recruitment of macrophages to corneal tissue of C57BL/6 mice infected by *A. fumigatus*.

### 3.5 Effect of up-/down-regulation of dectin-1 expression on macrophage polarization in mouse *A. fumigatus* keratitis

**Figure 5:**
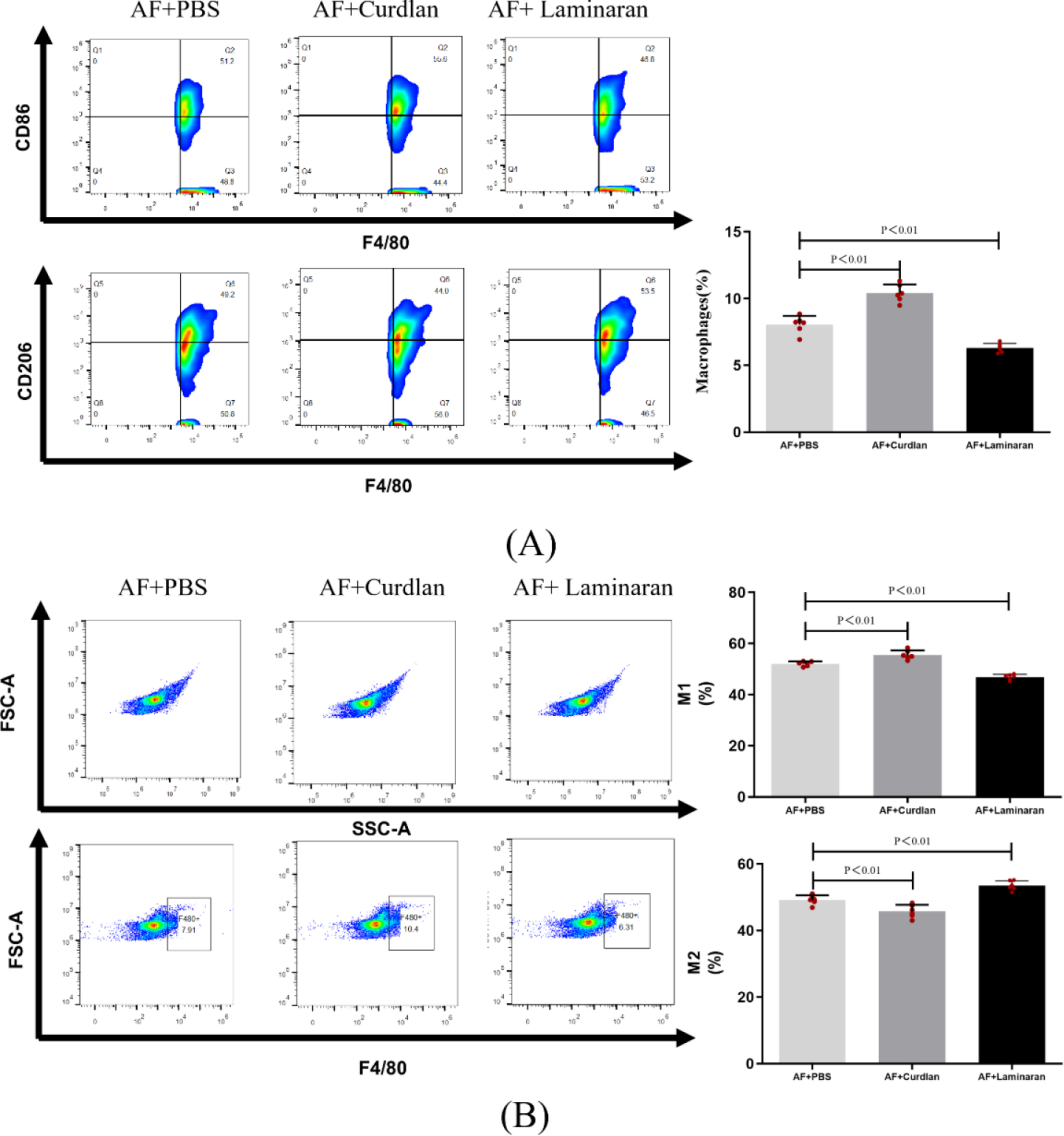
**(A)** Flow cytometry to detect changes in the number of macrophages in mouse corneas after 3 days of infection with up-/down-regulated Dectin-1 expression of *A*. *fumigatus*; **(B)** Flow cytometry to detect changes in the proportion of M1 and M2 type macrophage in mouse corneas after 3 days of infection with up-/down-regulated Dectin-1 expression of *A*. *fumigatus*. Macrophage labeling (CD45+F4/80+), M1 macrophage labeling (CD45+F4/80+CD86+), and M2 macrophage labeling (CD45+F4/80+CD206+) in the corneas of mice. Mouse corneas (6/group).

Flow cytometry results showed an increase in the number of macrophage in the mouse *A. fumigatus* model in the curdlan pretreated group (10.4%) and a decrease in the number of macrophage in the mouse *A. fumigatus* model in the laminaran pretreated group (6.31%), as compared to that in the AF+FBS group (7.91%), after 3 days of corneal *A. fumigatus* infection. Further flow cytometric analysis of M1-type macrophage labeled with CD45+ F4/80+CD86+ triple staining and M2-type macrophage labeled with CD45+ F4/80+CD206+ triple staining revealed an increased proportion of M1-type macrophage in the curdlan pretreated group (55.6%), compared to the AF+FBS group (51.2%). laminaran pretreatment group had a decreased proportion of M1-type macrophage (46.8%); whereas M2-type macrophage were the opposite of M1-type: the proportion of M2-type macrophage was 49.2% in the AF+FBS group, the proportion of M2-type macrophage was decreased in the curdlan pretreatment group (44.0%), and the proportion of M2-type macrophage was increased in the laminaran pretreatment group (53.5%) (**Figure 5**).

### 3.6 Effect of the MAPK signaling pathway on macrophage phenotype-related factors in RAW264.7 cells

**Figure 6:**
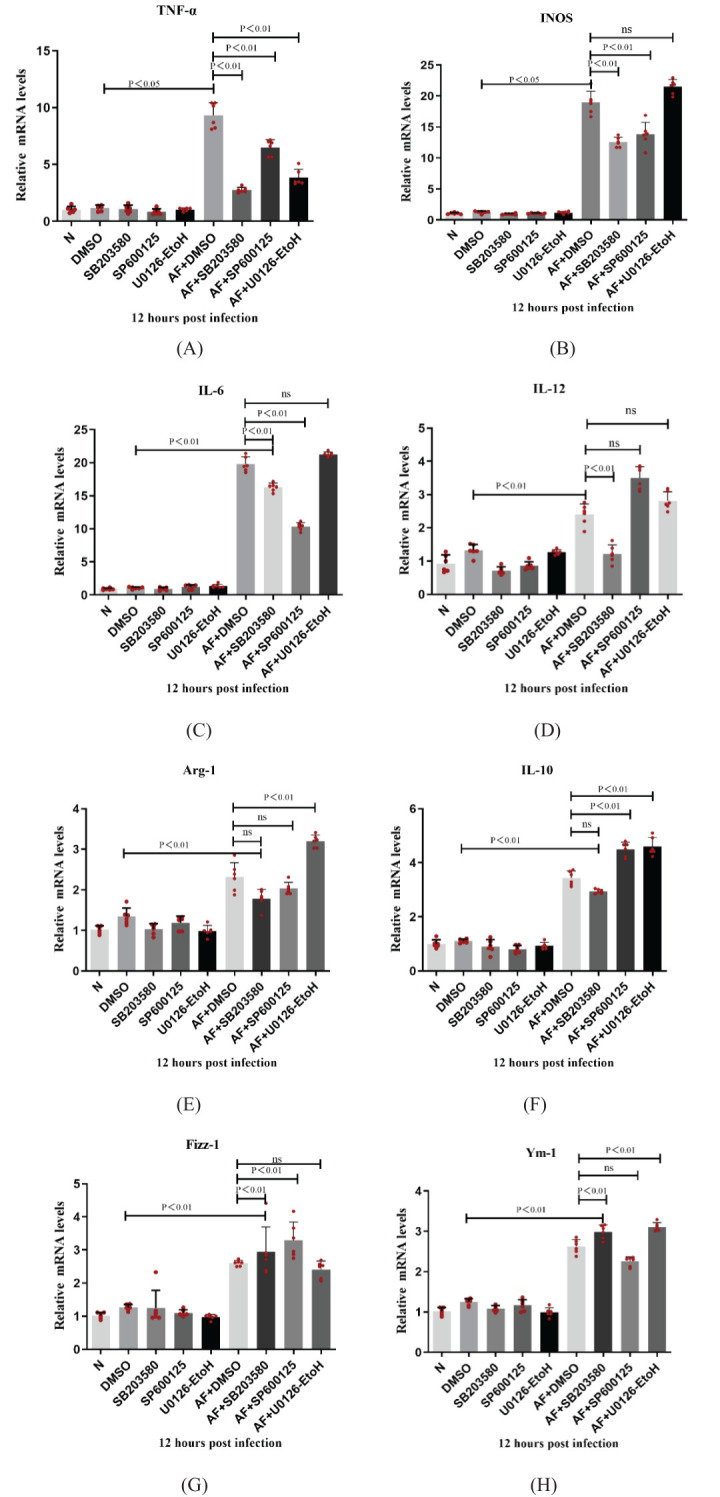
Dectin-1 regulated the macrophage polarization through the MAPK signaling pathway in the RAW264.7 cells. The expressions of TNF-α, INOS, IL-6, IL-12, Arg-1, IL-10, Fizz-1 and Ym-1 were examined by RT-PCR in RAW264.7 cells **(A-H)**. Values represent as mean ± SEM. RAW264.7 cells (6-hole plate/group).

RAW264.7 cells were pretreated with MAPK pathway inhibitors for 2 h, then stimulated with *A. fumigatus* for 12 h. Compared with the control group, the expression of M1, but not M2, cytokine mRNAs in the p38 inhibitor pretreatment group decreased significantly (P < 0.05). Compared with the control group, the JNK inhibitor pretreatment group had significantly decreased expression of M1 cytokine mRNAs (P < 0.05), while expression of IL-10 mRNA increased significantly (P < 0.05). Compared with the control group, the expression of TNF-α mRNA (M1 cytokine) in the ERK inhibitor pretreatment group decreased significantly (P < 0.05), while the expression of Arg-1 and IL-10 mRNAs increased significantly (P < 0.05). At the same time, the p38, JNK, and ERK inhibitor pretreatment groups showed no significant changes in mRNA expression levels of TNF-α, INOS, IL-6, IL-12, Arg-1, IL-10, Fizz-1 and Ym-1 compared with the normal group (**Figure 6**). From **Figure 6**, we can conclude that the MAPK cell signaling pathway is involved in macrophage polarization of RAW264.7 cells. Inhibition of MAPK cell signaling pathway can lead to increased expression of phenotype related factors in M2 macrophages.

## 4 Discussion

Fungal keratitis is a serious blinding corneal infectious disease that can lead to corneal opacity, and even blindness in severe cases (28). Innate immunity is the first line of defense for the body’s immune function. Dectin-1 is one of the pattern recognition receptors (29–31). Current studies have found that a variety of cell processes participate in the antifungal immune defense response through Dectin-1, including phagocytosis, induction of killing mechanisms, and promotion of the production of cytokines and chemokines (32, 33). In our study, mice pretreated with a Dectin-1 agonist exhibited a more severe corneal inflammatory reaction and higher inflammatory scores compared to the control group. Conversely, mice pretreated with a Dectin-1 inhibitor showed reduced corneal inflammation and lower inflammatory scores. These findings similar with Brown’s research (37, 38), which demonstrated that Dectin-1 promotes the aggregation of activated macrophages and cytokine production by recognizing fungal β-1,3-glucan, thereby enhancing resistance to Aspergillus infection.

In our experiment, we found that Dectin-1 can regulate the infiltration of macrophages in the cornea of mice infected with *Aspergillus fumigatus*, and more importantly, Dectin-1 can also affect macrophage polarization. Macrophage are key mediators of the innate immune response against foreign pathogens (including bacteria and fungi) (34–36). At present, macrophage are mainly divided into M1 (classically activated macrophage) and M2 (alternatively activated macrophage) classes, according to different activation states and functions (13). Linda et al. (37) in their study of Pseudomonas aerugINOSa keratitis found that in the early stage of infection, macrophage in the cornea were polarized mainly towards M1-type macrophage, whereas in the period of recovery from corneal ulceration, macrophage in the cornea were polarized mainly towards M2-type macrophage. These studies suggest that macrophages are involved in the process of corneal inflammatory response through polarization of different phenotypes at different times of infection. We also observed that the number of macrophages in the corneal stroma of mice in the fungal infection alone and Dectin-1 pretreatment groups increased significantly compared with the control group. In contrast, the number of macrophages in the Dectin-1 inhibitor pretreatment group decreased significantly. Together, these data indicate that macrophage, as important defense cells of the body, participate in the innate immune process, and Dectin-1 can affect the number of macrophages in the corneas of mice. Our research shows that Dectin-1 can affect macrophage recruitment and polarization in mouse *A. fumigatus* keratitis. In the early stage of *Aspergillus fumigatus* infection, macrophage polarized toward the M1 type, while influencing the expression of macrophage phenotype-associated factors through the MAPK signaling pathway to regulate the process of the immune-inflammatory response; on the contrary, in the late stage of the infection, macrophages were polarized toward the M2 type. Our experiments revealed that Dectin-1 could affect the phenotypic changes of corneal macrophage, activating Dectin-1 macrophage polarized towards M1 type, increased the secretion of pro-inflammatory factors, and aggravated the corneal inflammatory response, whereas when Dectin-1 was inhibited, macrophage polarized towards M2 type, inhibited the secretion of pro-inflammatory factors, promoted the secretion of inflammatory factors, and attenuated the corneal inflammatory response. It should be noted that the agonist curdlan treatment group did not cause significant corneal inflammation reactions, and there were no significant differences in corneal clinical scores and the number of macrophages in the cornea. (**Supplementary Figure 3S1**). However, the detection of inflammatory factors in the cornea partially increased, but significantly lower than the expression level in the fungal infection group. We consider that fungal invasion of the cornea may not only be caused by the cascade reaction of cell signaling through the binding of fungal cellwall component β - glucan to host membrane receptor Dectin-1, but also by the host toxin response caused by various toxins secreted by fungi, or it is possible that fungi can activate other pattern recognition receptors (TLR2, TLR4, LOX-1 ect). Walachowski S’s research shows that triggering Dectin-1-pathway alone is not sufficient to induce cytokine production by murine macrophages. β-glucans (zymosan, soluble glucan-enriched compound and curdlan) the composition of the cell wall saccharomyces cerevisiae is poor inducers of chemokine and cytokine production in murine macrophages (39). These results suggest that dectin-1 alone is not sufficient to trigger NFκB/AP-1 signaling in macrophages but crosstalk between dectin-1 and other PRRs, such as TLR2 and TLR4, greatly enhances NFκB-associated cytokine production. We demonstrated that the effect of the Dectin-1 agonist Curdlan on the experimental baseline in the supplementary data (**Supplementary Figure 3S2**). Compared with the control group, after 3 days of pretreatment with Dectin-1 agonist (Curdlan), only INOS, IL-6 expression of M1 phenotype related factors was slightly increased in the cornea of mice. Based on the results of our agonist curdlan pretreatment of fungal infections, we consider that agonist curdlan pretreatment only amplifies the corneal inflammation response after fungal infection.

Previous studies showed that the Dectin-1-Syk signaling pathway induced the activation of MAPKs in epithelial cells infected with Candida albicans (3, 40). In addition, a study by Bian et al. (41) showed that the MAPK/p38 signaling pathway promoted migration and the production of proinflammatory cytokines in macrophage activated by lipopolysaccharide. Sha et al. (21) found that the MAPK signaling pathway (including p38, JNK, and ERK) was involved in generating macrophage phenotype-related factors in a hypoxia-induced macrophage polarization model. Inhibition of MAPK pathways associated with M1 polarization might lead to increased M2 macrophage activity, potentially resulting in a reduced ability to combat *A. fumigatus*. Inhibition of MAPK pathways associated with M2 polarization might enhance the M1 response, potentially improving fungal clearance but also possibly leading to excessive inflammation.In summary, MAPK inhibition can significantly influence the M1/M2 macrophage balance in response to *A. fumigatus*, which in turn can affect the outcome of the infection and the effectiveness of the host’s immune response (42–44). Our study also found that after fungal infection, Dectin-1 affected the expression levels of phenotype-related factors (TNF-α, INOS, IL-6, IL-12, Arg-1, or IL-10) in M1 and M2-type macrophages through the regulation of MAPK signaling pathways, such as p38, JNK, and ERK. However, not all MAPK cell signaling pathways are involved in the expression changes of macrophage phenotype-associated factors in *A. fumigatus*, and at the same time do not regulate all M1/M2 type-associated factors. For instance, activation of ERK could enhance the expression of M2 markers such as IL-10 or ARG1 while not significantly affecting M1 markers like TNF-α or IL-12 (45). We did not investigate the expression changes of M1/M2 macrophage phenotype related proteins and specific targets after MAPK inhibition in this part of the experiment. Our experimental results in this part only indicate that MAPK may be involved in macrophage polarization.We will further investigate their changes and possible mechanisms in future experiments.

In summary, Dectin-1 can affect the macrophage recruitment and polarization in mouse corneal, regulate changes of macrophage M1 and M2 cytokines to participating in the process of corneal antifungal innate immunity through the MAPK signaling pathway in fungal keratitis. Our study enriches the mechanism related to the involvement of Dectin-1 in the intrinsic immunity against antifungal infection in *Aspergillus fumigatus* keratitis, and provides the basis for laboratory relevant data on Dectin-1 as a potential therapeutic target.

## Data Availability

The original contributions presented in the study are included in the article/**Supplementary Material**. Further inquiries can be directed to the corresponding author.
