# Supplementary material for: Dectin-1 participates in the immune-inflammatory response to mouse Aspergillus fumigatus keratitis by modulating macrophage polarization
Source: Front Immunol. 2024 Oct 16;15:1431633. doi: 10.3389/fimmu.2024.1431633 (PMC11523060; doi:10.3389/fimmu.2024.1431633)
Supplement: Supplementary file 1 [file DataSheet1.doc]

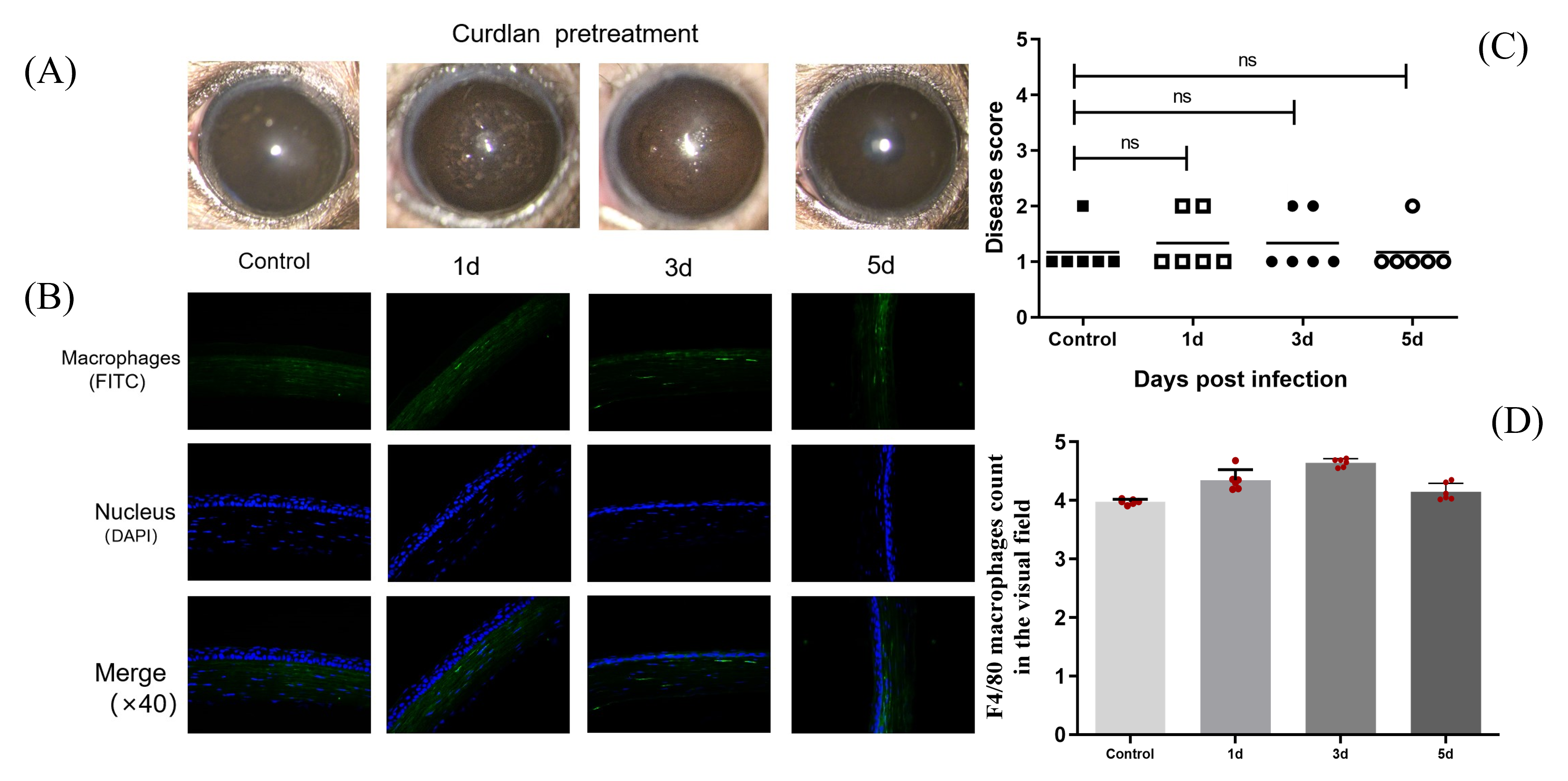


Figure3S1 Effects of pretreatment with Dectin-1 agonist Curdlan on anterior segment response and the number of macrophages in the cornea of mice. (A) Clinical signs and (B) clinical score in the mice cornea on day 1, day 3, and day 5 pretreated with Dectin-1 agonist compared to the control group corneas. (C) Macrophages in the mice cornea on day 1, day 3, and day 5 pretreated with Dectin-1 agonist compared to the control group corneas. (D) The comparison of macrophage count in the visual field of the corneas. Values represent as means ± SEM.


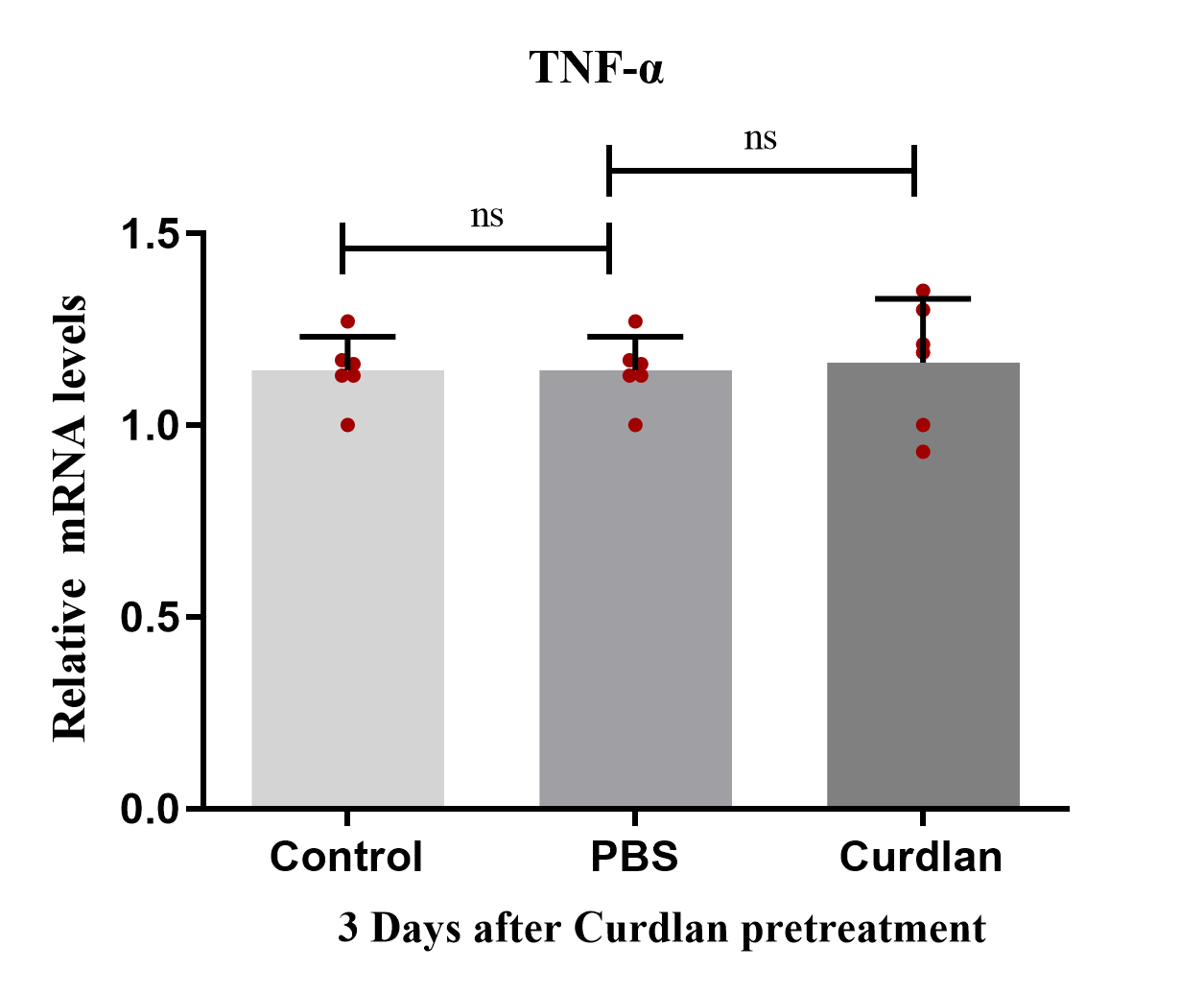

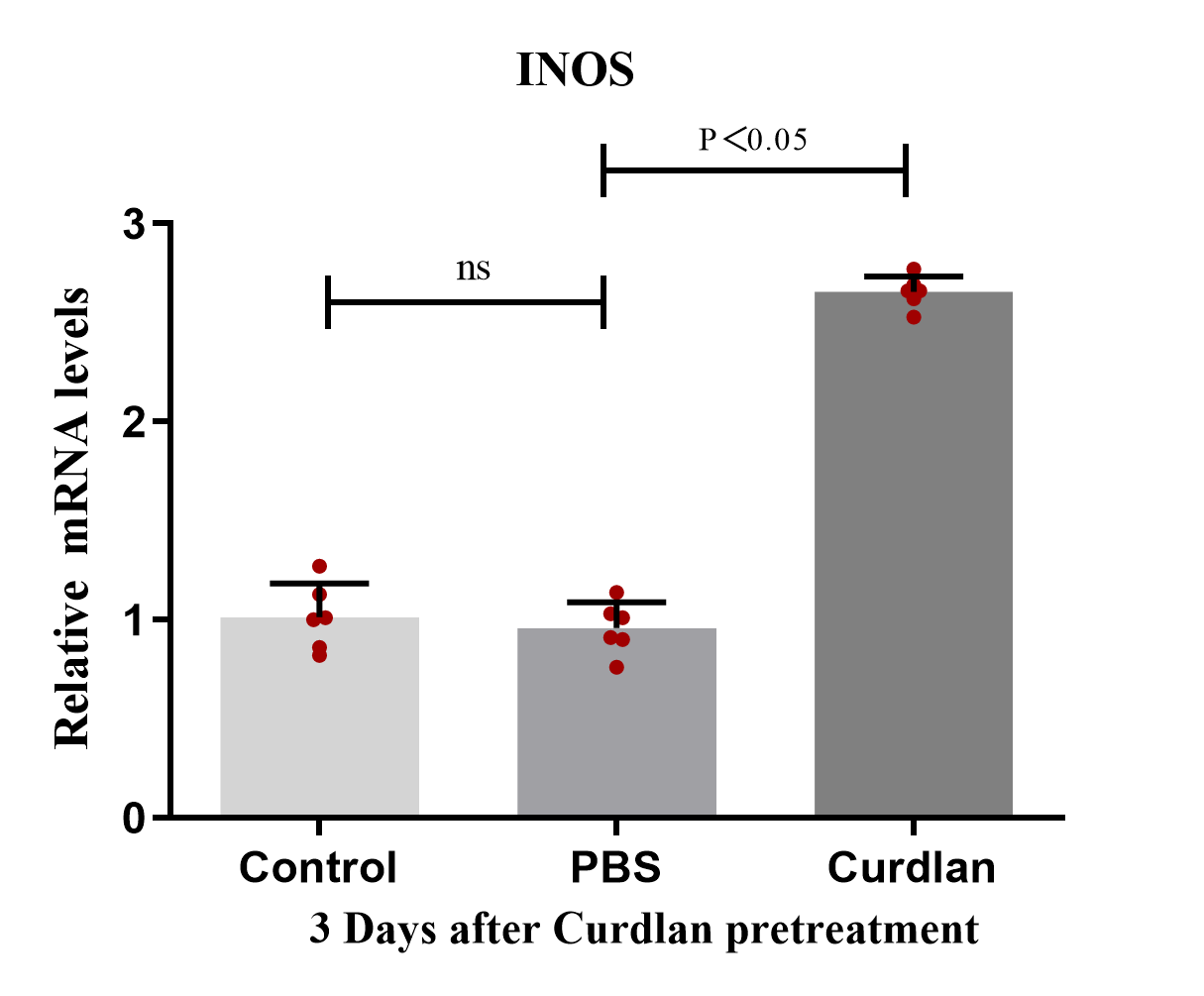


(A) (B)


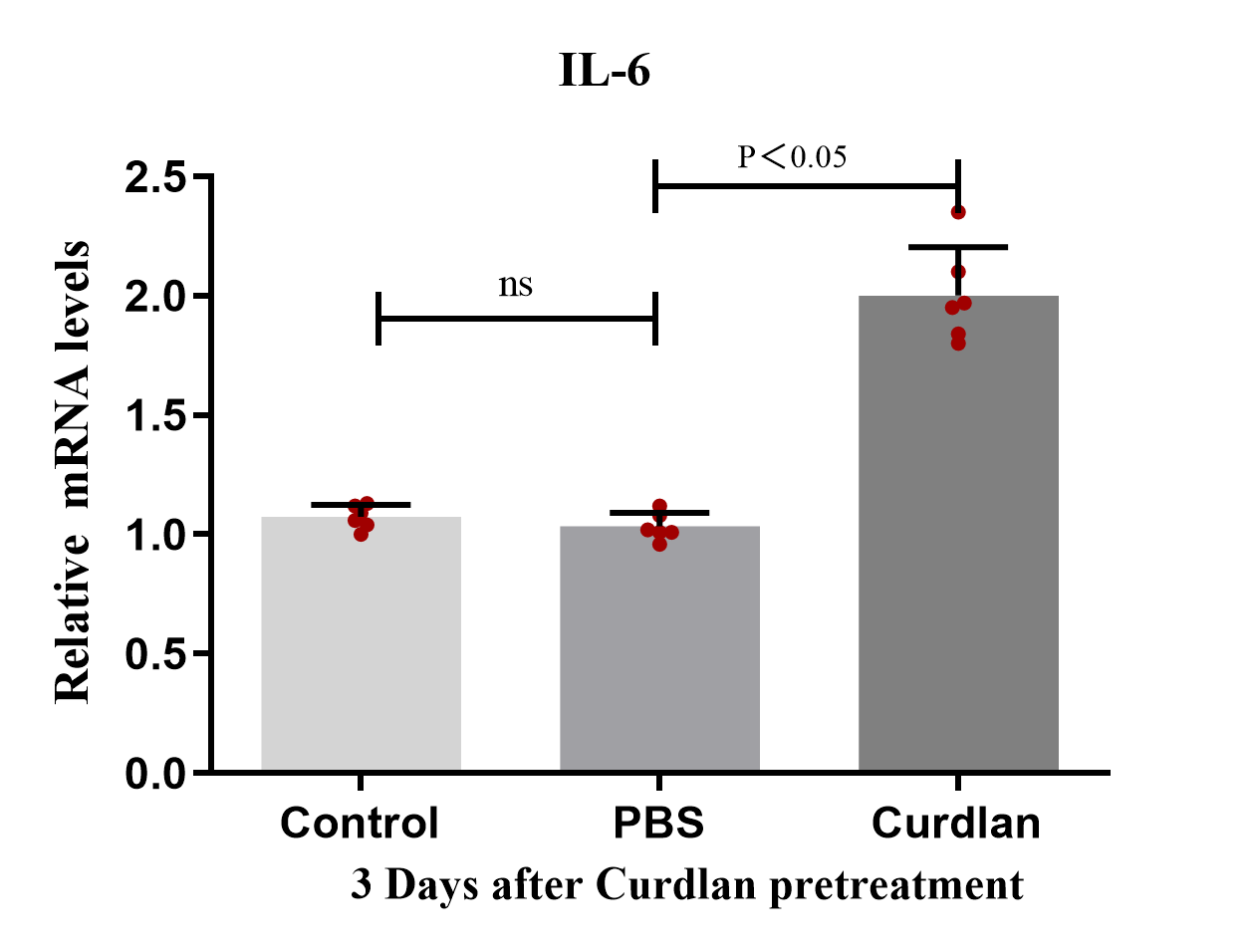

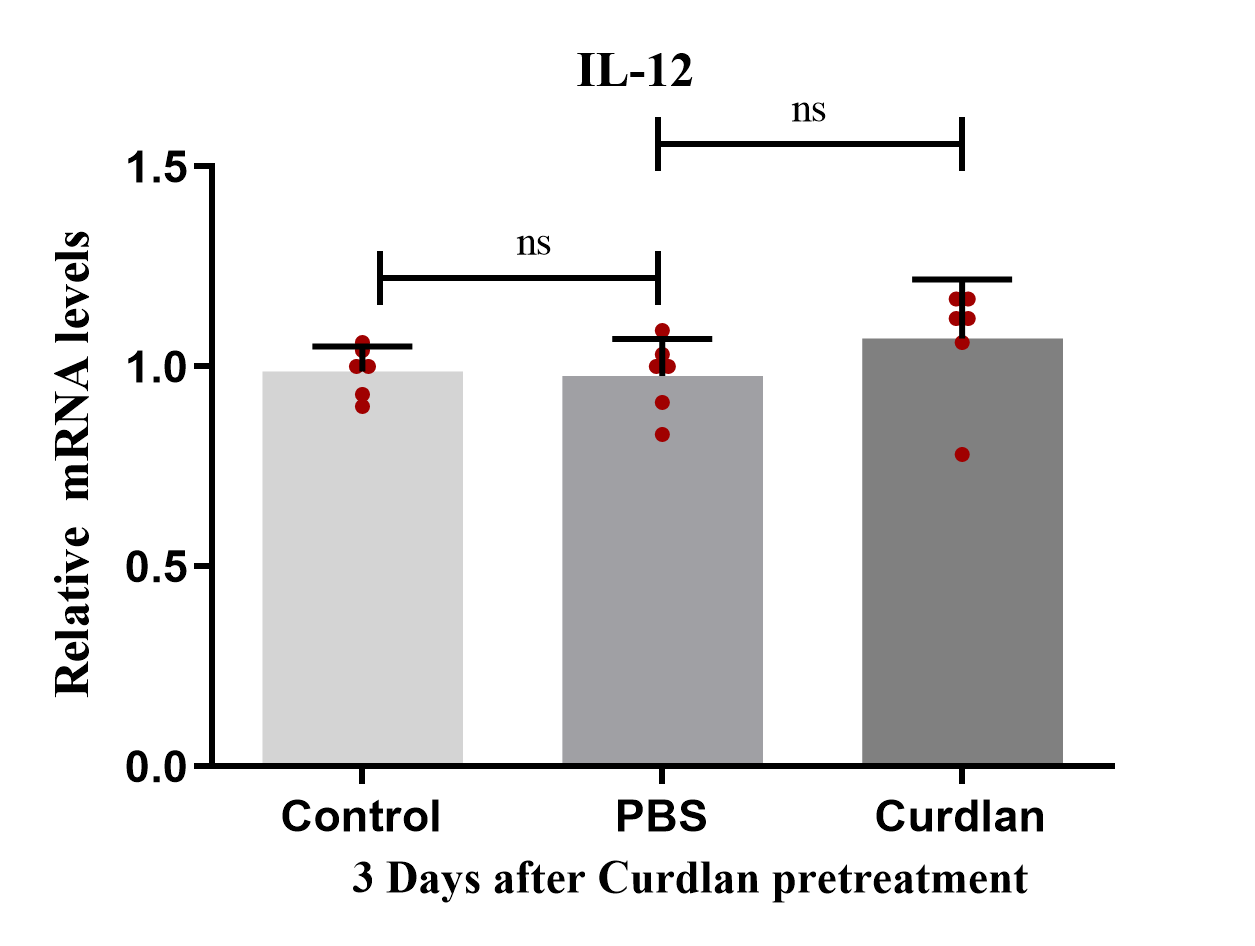


(C) (D)


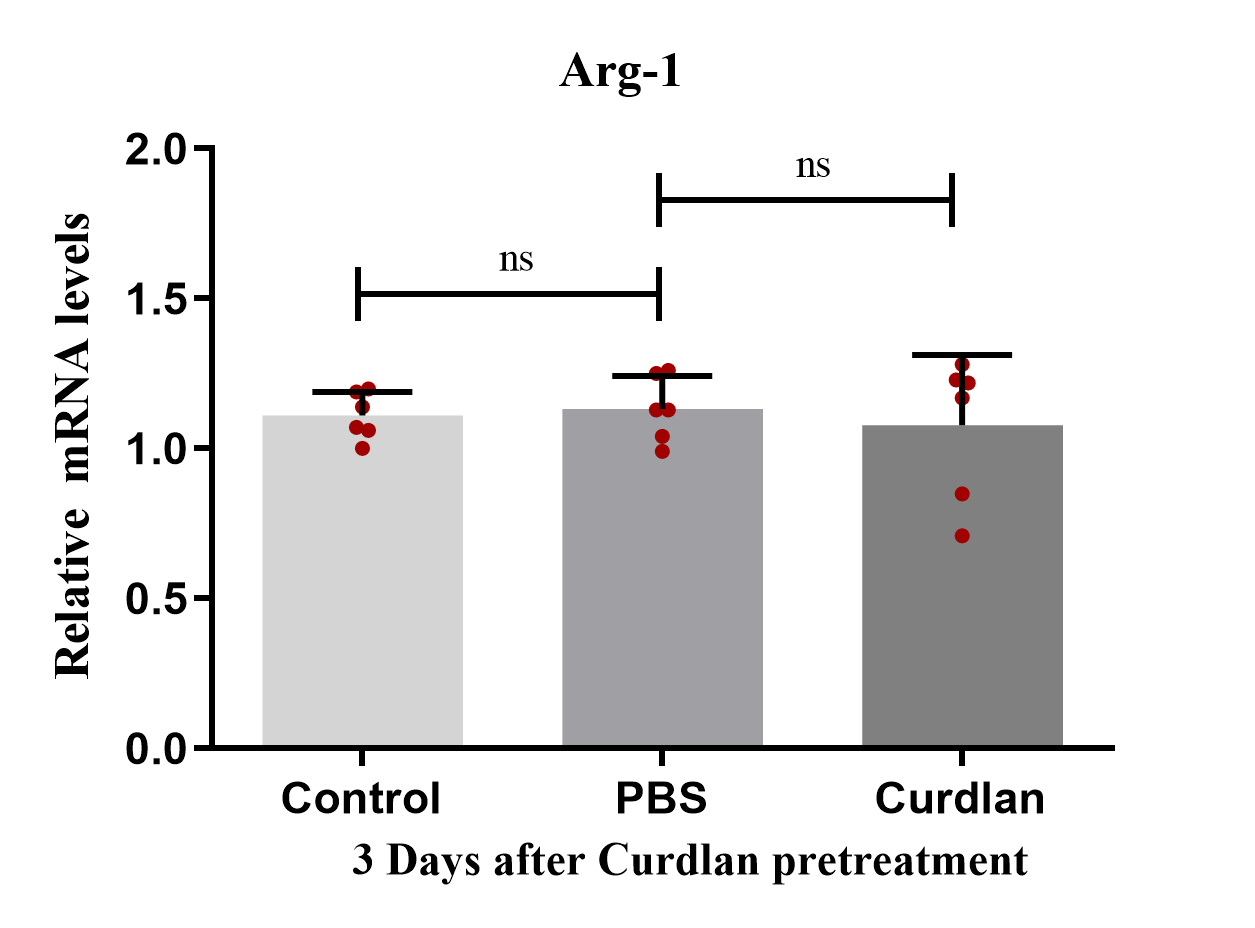

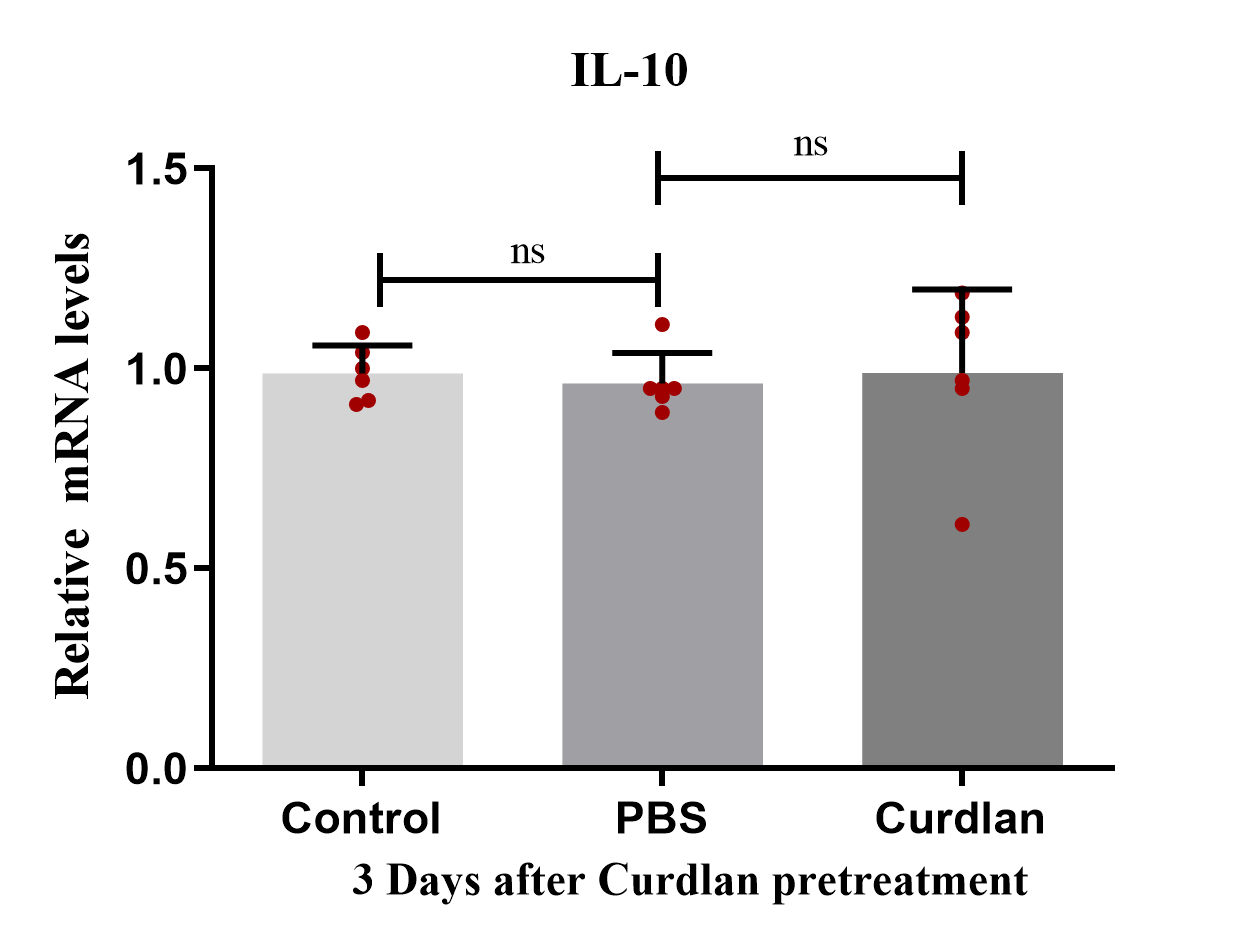


(E) (F)


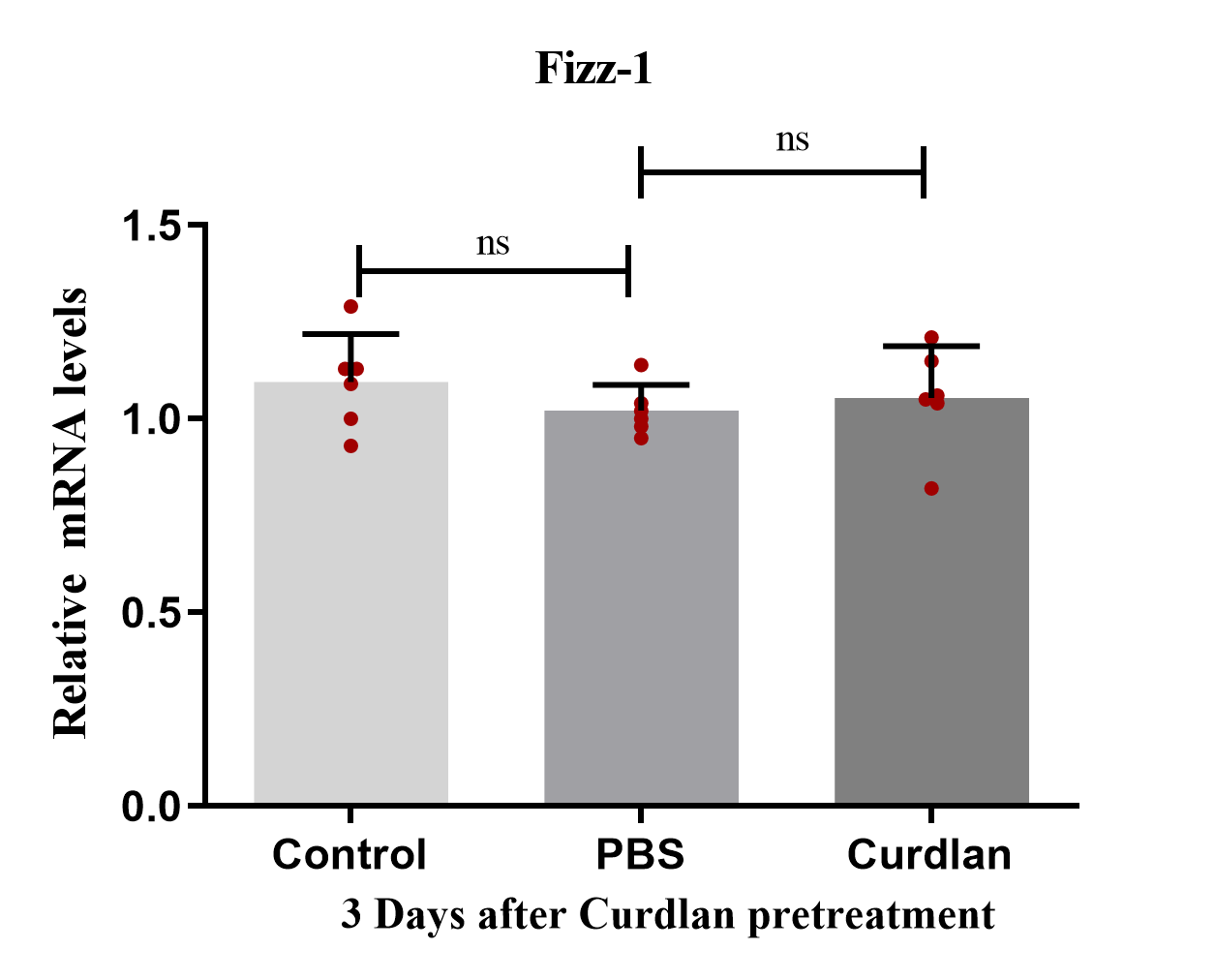

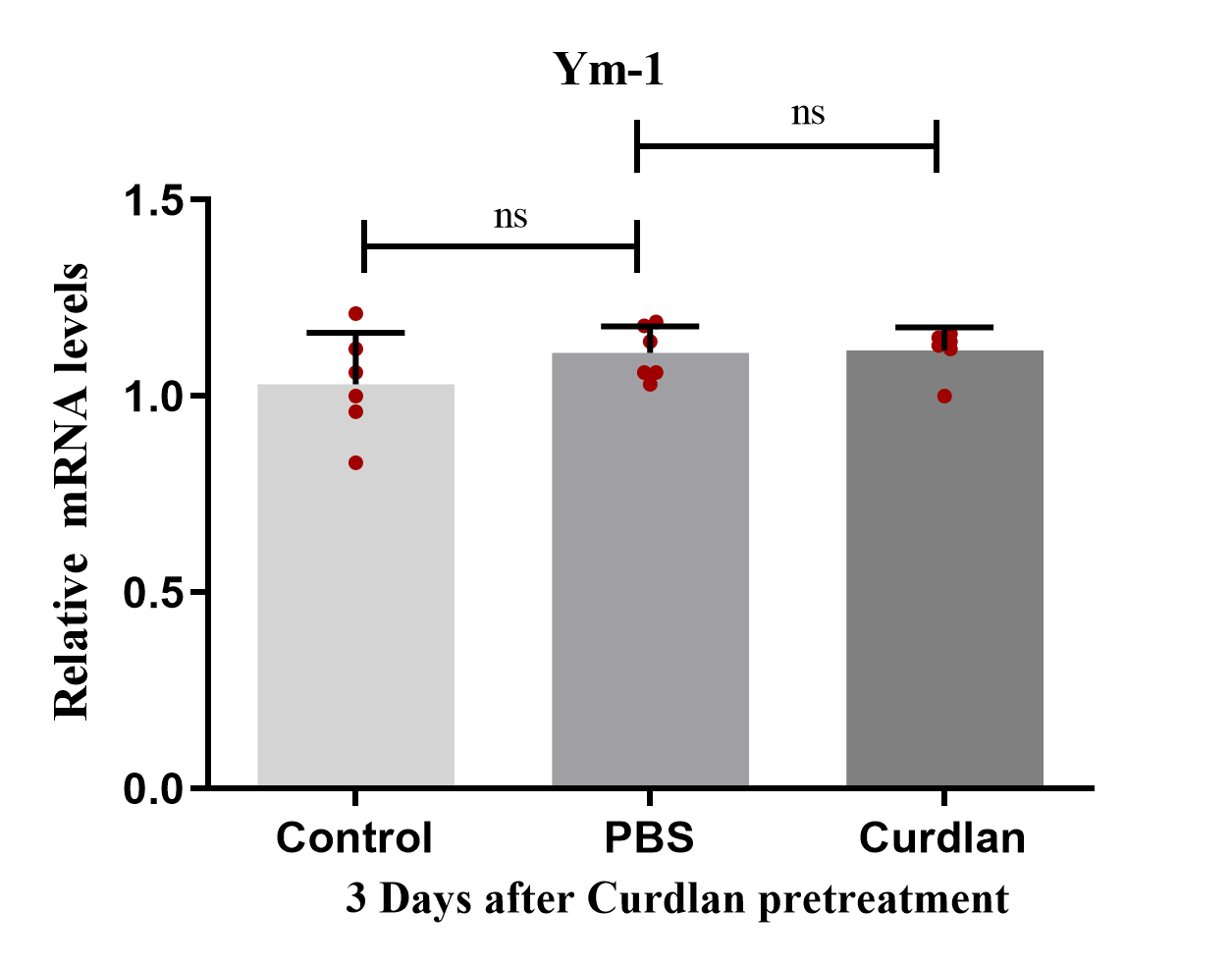


(G) (H)

Figure3S2. The expression levels of M1/M2 phenotype related factors mRNA in mouse cornea after 3 days of Dectin-1 agonist (Curdlan) pretreatment. Values represent as means ± SEM.


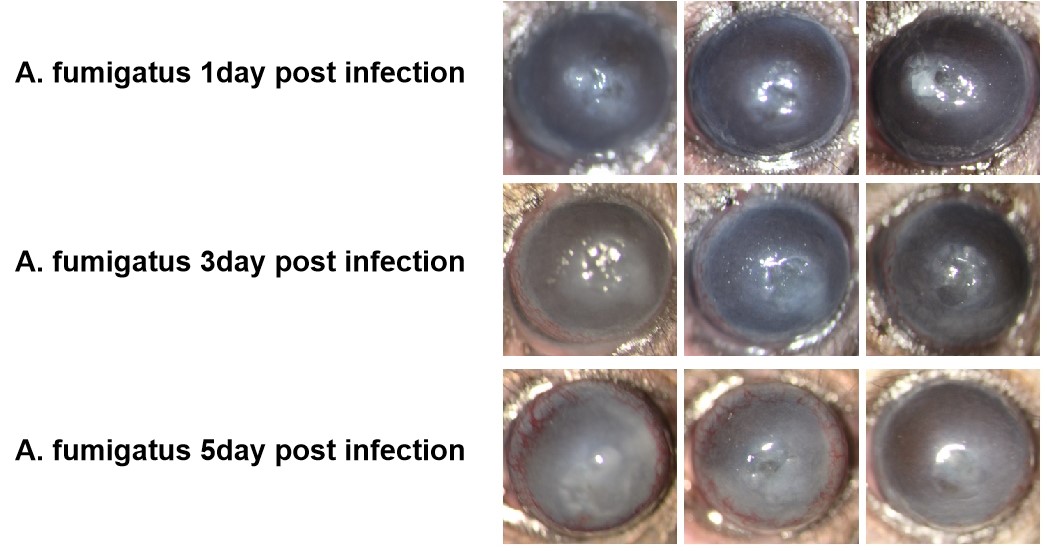


(A)


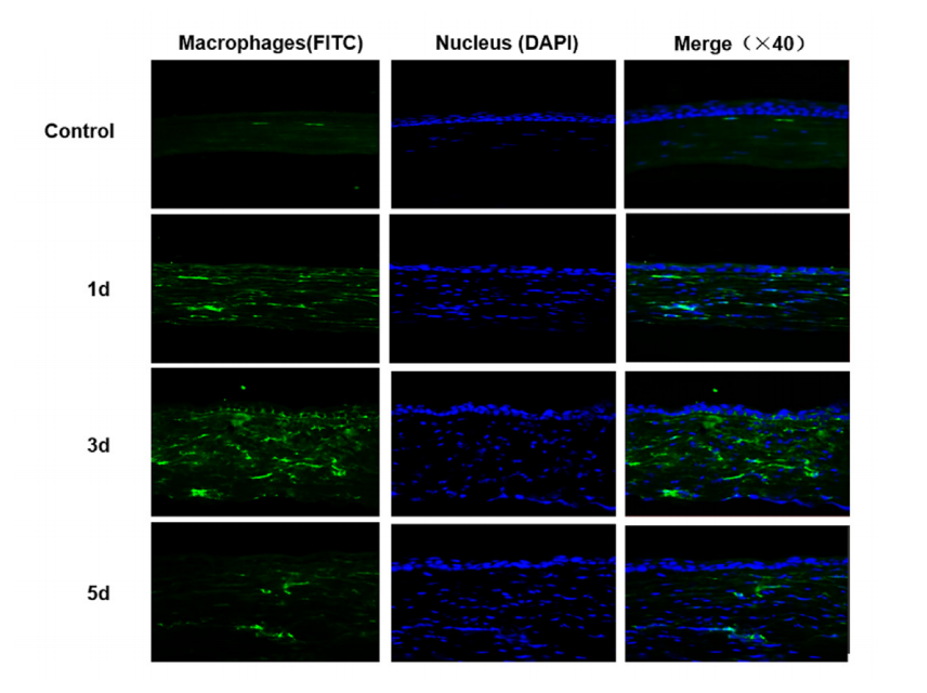


(B)

Figure5S (A)Clinical signs and (B) Macrophage infiltration of fungal keratitis in the mice cornea on day 1, day 3, and day 5 p.i. with A. fumigatus.
